# The Importance of Ventral Hippocampal Dopamine and Norepinephrine in Recognition Memory

**DOI:** 10.3389/fnbeh.2021.667244

**Published:** 2021-04-13

**Authors:** Joep Titulaer, Carl Björkholm, Kristin Feltmann, Torun Malmlöf, Devesh Mishra, Carolina Bengtsson Gonzales, Björn Schilström, Åsa Konradsson-Geuken

**Affiliations:** ^1^Section of Neuropsychopharmacology, Department of Physiology and Pharmacology, Karolinska Institutet, Stockholm, Sweden; ^2^Section of Neuropharmacology Addiction and Behavior, Department of Pharmaceutical Biosciences, Uppsala University, Uppsala, Sweden

**Keywords:** ventral hippocampus, hippocampus, dopamine, norepinephrine, microdialysis, novelty, novel object recognition (NOR), recognition memory

## Abstract

Dopaminergic neurons originating from the ventral tegmental area (VTA) and the locus coeruleus are innervating the ventral hippocampus and are thought to play an essential role for efficient cognitive function. Moreover, these VTA projections are hypothesized to be part of a functional loop, in which dopamine regulates memory storage. It is hypothesized that when a novel stimulus is encountered and recognized as novel, increased dopamine activity in the hippocampus induces long-term potentiation and long-term storage of memories. We here demonstrate the importance of increased release of dopamine and norepinephrinein the rat ventral hippocampus on recognition memory, using microdialysis combined to a modified novel object recognition test. We found that presenting rats to a novel object significantly increased dopamine and norepinephrine output in the ventral hippocampus. Two hours after introducing the first object, a second object (either novel or familiar) was placed in the same position as the first object. Presenting the animals to a second novel object significantly increased dopamine and norepinephrine release in the ventral hippocampus, compared to a familiar object. In conclusion, this study suggests that dopamine and norepinephrine output in the ventral hippocampus has a crucial role in recognition memory and signals novelty.

## Introduction

The dopaminergic neurons projecting from the ventral tegmental area (VTA) to the ventral hippocampus have been hypothesized to be part of a functional loop through which dopamine signals novelty and strengthens memory consolidation in the hippocampus (Lisman and Grace, 2005). This model proposes that the hippocampus detects novel information and signals *via* the subiculum and subcortical structures, to the VTA. Through activation of VTA dopamine cells, a novel stimulus would lead to increased dopaminergic activity in the hippocampus, which would activate postsynaptic dopamine receptors resulting in induction of long-term potentiation (LTP) and memory storage of the novel information.

In previous preclinical studies, Ihalainen et al. (1999) showed that placing an animal in a novel cage enhanced dopamine and norepinephrine release within the hippocampus. In addition, using 6-OH-DA lesions in catecholaminergic neurons in the hippocampus, Moreno-Castilla et al. (2017) showed that dopamine and norepinephrine output in the hippocampus is necessary for signaling novel spatial configurations, but do not affect recognition memory. Furthermore, Kempadoo et al. (2016) showed that optostimulation of dopamine transporter (DAT) or tyrosine hydroxylase (TH^+^) expressing neurons projecting from the locus coeruleus (LC) to the dorsal hippocampus improved spatial object recognition. Interestingly, the study by Kempadoo et al. (2016) suggest that dopamine is co-released from norepinephrine terminals of the LC. The authors conclude that the LC constitutes the main source of dopamine in the hippocampus. The expression of DAT in the hippocampus is scarce (Sesack et al., 1998; Borgkvist et al., 2012), and it has previously been hypothesized that in regions with low DAT concentrations, the dopaminergic transmission is regulated by norepinephrine terminals (Devoto et al., 2001). Consequently, both dopamine and norepinephrine are suggested to be cleared from the synaptic cleft by the norepinephrine transporter (NET). Accordingly, drugs blocking the NET have been found to also increase dopamine in the hippocampus (Borgkvist et al., 2012).

Previously we showed, that local activation of dopamine by the D_1/5_ receptor (D_1/5_R) agonist SKF81297 in the dorsal hippocampus, as well as the prelimbic region of the medial prefrontal cortex (mPFC), potentiates recognition memory (De Bundel et al., 2013). Moreover, a local injection of the D_1/5_R antagonist SCH23390 in the dorsal hippocampus or the mPFC prevented the formation of episodic-like memory in the novel object recognition (NOR) test (De Bundel et al., 2013). In a later study on recognition memory, we showed that rats recognize objects for 2 h post training, but not 24 h when two identical objects are presented for 2 min during the training session of the NOR test (Feltmann et al., 2015). The above-mentioned studies suggest that hippocampal dopamine signaling is involved in detecting novelty as well as spatial-and recognition memory.

Based on the model proposed by Lisman and Grace (2005), we hypothesize that any change in dopamine output that is observed when a novel object is presented compared to a familiar object, should be related to the detection of novelty in the hippocampus. Therefore, we measured dopamine and norepinephrine release in the ventral hippocampus using microdialysis when rats were exposed to novel-and familiar objects in the NOR test.

## Materials and Methods

### Animals

Male Wistar rats were obtained from Charles River (Germany) and had an average weight of 250 g upon arrival to the animal facility. The animals were kept under standard laboratory conditions with a 12/12 h light/dark cycle (lights on at 6 am), controlled temperature (21 ± 0.4°C) and relative humidity (55–65%) with *ad libitum* access to food (R34, Ewos Södertälje, Sweden) and water. The rats were allowed to acclimatize at least 5 days prior to any experiments. All experiments were performed in the light phase between 8 am and 6 pm. The experiments were approved by Karolinska Institutet and the local animal research ethics committee, Stockholm North.

### Microdialysis

Two days prior to the experiment, the rats were anesthetized intraperitoneally with a 5 ml/kg cocktail of Hyponorm^®^ (0.315 mg/ml fentanyl citrate and 10 mg/ml fluanisone; Janssen-Cilag, UK) and Dormicum^®^ (5 mg/ml midazolam; Roche AB, Sweden) diluted in purified water (1:1:2, 5 mg/kg). The rats were mounted in a sterotaxic frame (Koch, USA) and implanted with a concentric dialysis probe in the ventral hippocampus (AP −5.6, ML −4.8, DV −8.2) according to the atlas of Paxinos and Watson (1998). The dialysis probe was produced in-house with a semipermeable dialysis membrane (Filtral AN69, Hospal Industrie, France).

During the experiments the probes were continuously perfused with artificial cerebrospinal fluid (containing in mM; 147 NaCl, 3 KCl, 2.2 CaCl_2_, 1.0 MgCl_2_, 1.0 NaHPO_4_ and 0.2 NaH_2_PO_4_, pH 7.4) at a flow rate of 2.5 μl/min and each sample was collected over 30 min (i.e., 75 μl). Dopamine and norepineprhine output were continuously measured in the ventral hippocampus. The samples were automatically loaded and injected into a reverse-phased high performance liquid chromatography system with a C-18 column (Kinetex 150 × 4.6 mm, 2.6 μM, Phenomenex, Torrance, CA, USA) controlled by a computerized system (Totalchrome WS, version 6.3, Perkin Elmer, USA). The mobile phase for separation of dopamine, norepinephrine and metabolites consisted of 55 mM sodium acetate buffer (pH 4.0) with 12% methanol and 0.55 mM octanesulfonic acid. Dopamine, norepinephrine and their metabolites were quantified online by sequential oxidation and reduction using electrochemical detection in a highly sensitive analytical cell (model 5011; ESA Bioscience, USA), which was controlled by a potentiostat (Cholochem II model 5200; ESA Bioscience) with a detection-limit of approximately 0.3 nM/L. A potential of −200 or 400 mV was applied to detect monoamines and their metabolites respectively. All chromatograms were recorded digitally. An external standard of dopamine and norepinephrine was used in order to calculate the dopamine and norepinephrine output. The experiment started when the rats showed a stable dopamine and norepinephrine release (<10% variation). Baseline values (100%) were calculated as the average of the two values prior to the introduction of an object. A separate baseline was calculated for the first object and the second object (novel or familiar).

After the experiment, the animals were sacrificed, and the brains were preserved in a 25% sucrose and 4% formaldehyde solution. The probe placement was verified microscopically in Nissl stained sections (50 μm). Only animals with correct probe placement were included in the study.

### Novel Object Recognition

**Figure 1:**
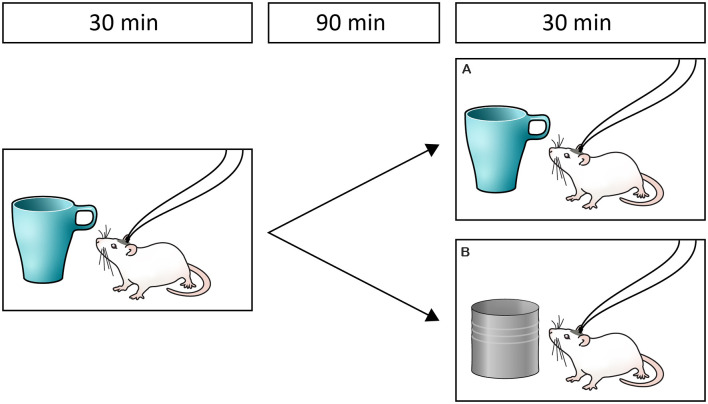
Experimental design. After steady baselines of dopamine and norepinephrine output were obtained, rats were presented with an object for 30 min and after a 90 min retention period, presented with either a familiar **(A)** or a novel object **(B)**.

The NOR test was first described in a article by Ennaceur and Delacour (1988). In this article, they presented two identical objects to rats and, after a retention period, the rats were presented a novel- and a familiar object. The time the animals explored the novel object compared to the familiar one was taken as a measure of the recognition memory of the animal (Ennaceur and Delacour, 1988). Due to the low extracellular dopamine concentration in the hippocampus and the relatively low temporal resolution of microdialysis, we modified our previously used NOR test (Feltmann et al., 2015). After steady baselines of dopamine and norepinephrine output were obtained (<10%), the rats were picked up while an object (a cup or a metal container) was placed in the experimental cage. The object was in the cage for the whole duration of the microdialysis sampling time (30 min). Two hours later, the rats were lifted again and a replica of the familiar object or a novel object was placed in the cage (Figure 1). All objects were cleaned with 70% ethanol before used in further experiments.

### Statistical Analysis

The absolute differences in basal dopamine and norepinephrine output in the ventral hippocampus between groups were analyzed using *t*-tests. The effect of an object on relative dopamine and norepinephrine output was compared to the last preceding sample using paired *t*-tests. The comparison between groups (novel vs. familiar) of the relative dopamine and norepinephrine release after the introduction of the second object was evaluated using *t*-tests. In all statistical evaluations *p* < 0.05 was considered statistically significant. All *p*-values were false discovery rate (FDR)-corrected for multiple comparisons.

## Results

### Absolute Baseline Values of Dopamine and Norepinephrine

**Figure 2:**
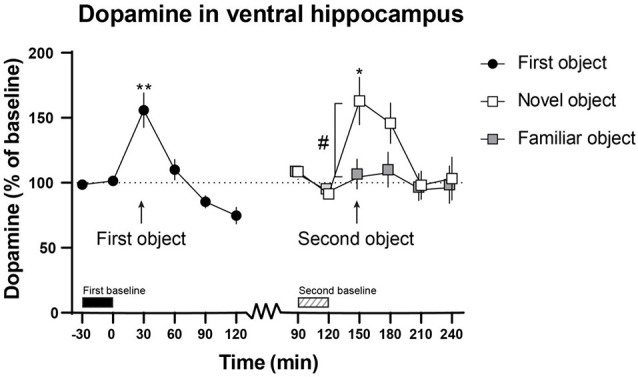
The effect of the first novel and familiar object on dopamine output in the ventral hippocampus. The first object and the novel object enhanced dopamine output in the ventral hippocampus. Moreover, the novel object enhanced dopamine output compared with the familiar object. The first object was presented to the rats from 0 to 30 min and the second object from 120 to 150 min. The time points used for the first (−30 to 0 min) and second (90–120 min) baseline are shown above the *x*-axis. *p < 0.05, **p < 0.01 vs. preceding baseline, ^#^*p* < 0.05 between novel and familiar objects. All *p*-values were false discovery rate (FDR)-corrected for multiple comparisons. *n* = 13, six for novel object and seven for familiar object in the ventral hippocampus.

**Figure 3:**
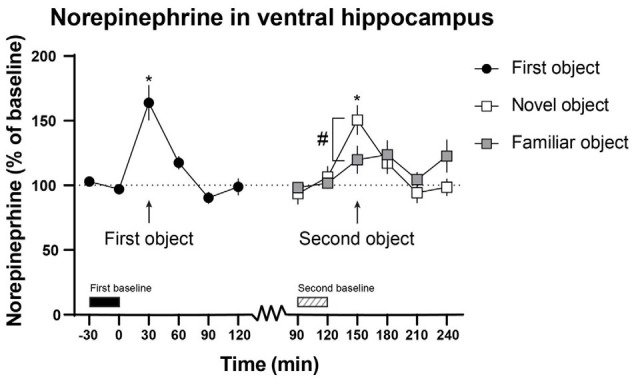
The effect of the first, novel and familiar object on norepinephrine output in the ventral hippocampus. The first and the novel object significantly increased norepinephrine output in the ventral hippocampus. Moreover, the norepinephrine output in the ventral hippocampus was significantly higher in the rats presented with the novel object compared with the rats presented with the familiar object. The first object was presented to the rats from 0 to 30 min and the second object from 120 to 150 min. The time points used for the first (−30 to 0 min) and second (90–120 min) baseline are shown above the *x*-axis. *p < 0.05 vs. preceding baseline, ^#^*p* < 0.05 between novel and familiar objects. All *p*-values were false discovery rate (FDR)-corrected for multiple comparisons. *n* = 13, six for novel object and seven for familiar object in the ventral hippocampus.

As the baselines shifted over time, and in order to compare the dopamine and norepinephrine output when the novel or familiar object was present to the preceding sample, separate baseline values were calculated for when the animals were introduced to the first object and a familiar or a novel object. The baselines were calculated from the two values 30 min before the objects were introduced to the animal (first object; −30–0 min, second object; 90–120 min, see Figures 2, 3).

The mean dopamine release (presented as mean ± SEM) during the first baseline in the ventral hippocampus was 0.191 ± 0.029 fmol/min. At the second baseline the mean dopamine release in the ventral hippocampus (*n* = 7) was 0.157 ± 0.046 fmol/min for the rats that got a familiar object and 0.110 ± 0.031 fmol/min for the rats that got a novel object (*n* = 6).

The mean norepinephrine output was 1.13 ± 0.255 fmol/min in the ventral hippocampus. At the second baseline the mean norepinephrine output in the ventral hippocampus was 0.963 ± 0.346 fmol/min for the rats that got a familiar object and 1.169 ± 0.371 fmol/min for the rats that got a novel object.

### Novel Object Recognition Combined With Microdialysis

After steady baselines of dopamine and norepinephrine were obtained, the animals were presented to the first object. Introduction of the first, novel object significantly increased ventral hippocampal dopamine output (155.8 ± 13.4%) compared with baseline values (101.4 ± 2.8%; *t*_(12)_ = 4.320, *p* = 0.008; Figure 2).

Two hours after the introduction of the first object (i.e., at 120 min), the second object (either novel or familiar) was introduced to the animal. The introduction of a novel object induced a significant increase of dopamine (163.0 ± 18.4%) output in the ventral hippocampus compared with the second baseline (91.5% ± 3.3; *t*_(5)_ = 3.591, *p* = 0.028; Figure 2). There was no difference in dopamine release in the ventral hippocampus when the familiar object was presented (104.6 ± 11.5%) compared with the second baseline (93.3 ± 4.2%). Furthermore, the dopamine output in the ventral hippocampus was significantly higher in the rats that were presented with a novel object (163.0 ± 18.4%) than in the rats that were presented with a familiar object (104.6 ± 11.5%; *t*_(11)_ = 2.775, *p* = 0.028; Figure 2).

The norepinephrine output in the ventral hippocampus (163.9 ± 13.6%) was significantly higher when the first object was presented than it was at baseline (97.0 ± 3.1%; *t*_(12)_ = 4.269, *p* = 0.027; Figure 3).

The introduction of a novel object induced an increase of norepinephrine (138.5 ± 11.2%) output in the ventral hippocampus compared with the second baseline (101.0 ± 8.2%; *t*_(5)_ = 3.089, *p* = 0.036; Figure 3). The rats that were presented with a novel object (143.7 ± 9.5%) had a significantly higher norepinephrine output in the ventral hippocampus compared with the rats that were presented with a familiar object (108.6 ± 5.1%; *t*_(11)_ = 3.379, *p* = 0.024; Figure 3).

## Discussion

In the present study, we show that dopamine and norepinephrine release in the rat ventral hippocampus increased in response to novelty. Therefore, our data lend support to the hypothesis proposed by Lisman and Grace (2005); suggesting that dopamine signals novelty in the hippocampus and thereby facilitates the formation of long-term memories.

The results presented here are also consistent with the findings from a study using object location memory combined to microdialysis in rats, which shows that novelty increased dopamine in the dorsal hippocampus (Moreno-Castilla et al., 2017). Moreover, our data are in line with a previous study showing that hippocampal norepinephrine is necessary for object recognition memory consolidation (Mello-Carpes et al., 2016). In addition, a recent study in rats also shows that memory persistence can be enhanced with physical exercise, which is completely blocked by an intra-hippocampal infusion of the D_1/5_R antagonist SCH23390, indicating that memory persistence is a dopamine dependent process (Vargas et al., 2020).

Furthermore, recent studies suggest that dopamine in the hippocampus may be co-released from norepinephrine neurons originating from the LC (Kempadoo et al., 2016; Takeuchi et al., 2016). Takeuchi et al. (2016) demonstrated that optostimulation of TH^+^ neurons projecting from the LC to the hippocampus improved spatial learning in a dopamine receptor dependent manner. Moreover, Kempadoo et al. (2016) show that there are significantly more dopamine containing neurons in the LC projecting to the dorsal hippocampus compared to the VTA. On the other hand, two separate studies have shown that novelty increased cell firing of dopamine cells in the VTA of both mice and cats (Horvitz et al., 1997; McNamara et al., 2014), supporting the notion that novelty-induced dopamine may originate from the VTA. However, in our study, it is not possible to draw any conclusion regarding the source of dopamine in the hippocampus, which could be the VTA and/or the LC.

In the present study, dopamine and norepinephrine release in the ventral hippocampus was increased in a similar way as described by Ihalainen et al. (1999). They found that dopamine and norepinephrine increased in the ventral hippocampus and mPFC when mice were handled or put in a novel cage. This could indicate that the observed dopamine and norepinephrine increase is caused by picking the rat up. However, in our study, there was a significant difference in dopamine and norepinephrine release between the rats that were presented to the novel object and the rats that were presented to the familiar object. Therefore, it seems that increase in dopamine and norepinephrine output must be related to the novelty of the object.

The observed dopamine and norepinephrine output in the ventral hippocampus is in line with a previous study, in which we showed that local administration of the NET inhibitor reboxetine strongly increased hippocampal dopamine release. In contrast, local administration of the DAT inhibitor GBR12909 only caused a negligible increase in dopamine release. Moreover, autoradiography revealed very low levels of DAT expression in the hippocampus. Thus, demonstrating that the dopamine and norepinephrine systems in the hippocampus have a shared uptake mechanism at the NET level (Borgkvist et al., 2012). In our earlier study (De Bundel et al., 2013), a local injection of D_1/5_R agonist SKF81297 in the mPFC or dorsal hippocampus potentiated recognition memory. Similarly, a local injection of D_1/5_R antagonist SCH23390 in the dorsal hippocampus or mPFC prevented the formation of episodic-like memory. It should be noted that in the present experiments, the objects were presented within 2 h, thus not involving long-term memories. Therefore, comparisons between the present results and previous behavioral data would be speculative.

It has previously been shown that other neurotransmitters besides dopamine and norepinephrine play an essential role in recognition memory. Stanley et al. (2012) combined microdialysis to novel object recognition and found an increased release of acetylcholine in the CA1 region of the ventral hippocampus when a familiar or a novel object was introduced to the animal. Interestingly, glutamate in the CA1 region was only increased when a novel object was presented to the animal, while the γ-aminobutyric acid (GABA) output was unaffected when introducing any of the objects. In agreement with these findings, Ihalainen et al. (2010) showed that acetylcholine release in the dorsal hippocampus was increased during an object recognition task. Using a similar methodology but in a different region of the hippocampus as Stanley et al. (2012); Moreno-Castilla et al. (2017) report no increase of either glutamate or GABA in the dorsal hippocampus. The above-mentioned studies indicate that there are delicate neurochemical differences within the hippocampal regions and that multiple neurotransmitters synergistically regulate memory formation.

Finally, by utilizing a version of our previously validated NOR test (Feltmann et al., 2015), in which we consecutively presented two novel objects or reintroduce a familiar object to the rat, we have demonstrated the feasibility of measuring dopamine and norepinephrine output in response to novelty. Thus, in the future, this paradigm can be used with other methods with higher temporal resolution to further study, the temporal aspects of neurotransmitter output in detection and response to novelty. Moreover, it would be of great interest to study the source of the dopamine and norepinephrine output in the ventral hippocampus. In this way, the specific roles of dopamine and norepinephrine could be investigated.

In conclusion, the present study provides support for the hypothesis that dopamine in the hippocampus signals novelty and suggests that a crucial role for ventral hippocampal dopamine and norepinephrine in recognition memory.

## Data Availability Statement

The raw data supporting the conclusions of this article will be made available by the authors, without undue reservation.

## Ethics Statement

The animal study was reviewed and approved by Karolinska Institutet and the local animal research ethics committee, Stockholm North.

## Author Contributions

JT analyzed the data, analyzed the experiments, made figures, and wrote the first draft.

CB performed and analyzed the experiments. KF, TM, DM, and CBG performed experiments. BS and ÅK-G led the research, designed experiments and wrote the manuscript. All authors contributed to the article and approved the submitted version.

## Conflict of Interest

CB is currently employed by Janssen-Cilag AB, but contributed to this study in his prior role at Karolinska Institutet. Janssen had no part in the development, execution or financing of this study. The remaining authors declare that the research was conducted in the absence of any commercial or financial relationships that could be construed as a potential conflict of interest.
